# The activity of circulating dipeptidyl peptidase-4 is associated with subclinical left ventricular dysfunction in patients with type 2 diabetes mellitus

**DOI:** 10.1186/1475-2840-12-143

**Published:** 2013-10-07

**Authors:** Susana Ravassa, Joaquín Barba, Isabel Coma-Canella, Ana Huerta, Begoña López, Arantxa González, Javier Díez

**Affiliations:** 1 Division of Cardiovascular Sciences, Centre for Applied Medical Research, University of Navarra, Pamplona, Spain; 2 Department of Cardiology and Cardiac Surgery, University of Navarra Clinic, University of Navarra, Pamplona, Spain; 3 Department of Internal Medicine, University of Navarra Clinic, University of Navarra, Pamplona, Spain

**Keywords:** Dipeptidyl peptidase-4, Type 2 diabetes mellitus, Subclinical left ventricular dysfunction

## Abstract

**Background:**

Patients with type 2 diabetes mellitus (T2DM) present subclinical left ventricular systolic and/or diastolic dysfunction (LVD). Dipeptidyl peptidase-4 (DPP4) inactivates peptides that possess cardioprotective actions. Our aim was to analyze whether the activity of circulating DPP4 is associated with echocardiographically defined LVD in asymptomatic patients with T2DM.

**Methods:**

In this cross-sectional study, we examined 83 T2DM patients with no coronary or valve heart disease and 59 age and gender-matched non-diabetic subjects. Plasma DPP4 activity (DPP4a) was measured by enzymatic assay and serum amino-terminal pro-brain natriuretic peptide (NT-proBNP) was measured by enzyme-linked immunosorbent assay. LV function was assessed by two-dimensional echocardiographic imaging, targeted M-mode recordings and Doppler ultrasound measurements. Differences in means were assessed by t-tests and one-way ANOVA. Associations were assessed by adjusted multiple linear regression and logistic regression analyses.

**Results:**

DPP4a was increased in T2DM patients as compared with non-diabetic subjects (5855 ± 1632 vs 5208 ± 957 pmol/min/mL, p < 0.05). Clinical characteristics and echocardiographic parameters assessing LV morphology were similar across DPP4a tertiles in T2DM patients. However, prevalence of LVD progressively increased across incremental DPP4a tertiles (13%, 39% and 71%, all p < 0.001). Multivariate regression analysis confirmed the independent associations of DPP4a with LVD in T2DM patients (p < 0.05). Similarly, multiple logistic regression analysis showed that an increase of 100 pmol/min/min plasma DPP4a was independently associated with an increased frequency of LVD with an adjusted odds ratio of 1.10 (95% CI, 1.04 to 1.15, p = 0.001).

**Conclusions:**

An excessive activity of circulating DPP4 is independently associated with subclinical LVD in T2DM patients. Albeit descriptive, these findings suggest that DPP4 may be involved in the mechanisms of LVD in T2DM.

## Introduction

Diabetic cardiomyopathy is a common but frequently unrecognized pathological process already present in asymptomatic patients with type 2 diabetes mellitus (T2DM) [1]. The left ventricle (LV) of these patients is characterized by an excessive growth that progresses from a normal functional state to asymptomatic LV dysfunction (LVD) [2,3]. In fact, the prevalence of subclinical LVD in patients with T2DM has been shown to vary from 25% to 60% in different studies [4-6]. Both subclinical LV diastolic dysfunction (LVDD) and subclinical LV systolic dysfunction (LVSD) have been described in the earliest phases of diabetic cardiomyopathy [7-9]. Importantly, recent studies demonstrate that subclinical LVD progress over 5 years in patients with T2DM despite improved glycemic control [10]. This may explain that T2DM is associated with increased risk of new-onset heart failure (HF) [11].

Dipeptidyl peptidase-4 (DPP4) is a membrane glycoprotein with serine peptidase activity located on the surface of various cell types, that may also exist in plasma and other body fluids as a soluble form lacking the cytoplasmic and transmembrane domains [12]. DPP4 is widely known for its role in regulation of glycemia through catabolism of the incretin glucose-dependent insulinotropic polypeptide (GIP) and glucagon-like peptide 1 (GLP-1), responsible for glucose-dependent insulin secretion from the pancreas [13]. A number of observations suggest that GLP-1 may influence the cardiovascular system [14] and exert direct cardiac protective actions through the interaction with its receptor localized in cardiac cells [15,16]. In this regard, a number of experimental evidences suggest that DPP4 inhibitors may provide cardioprotective actions beyond their glucose lowering effect [17]. For instance, it has been reported that DPP4 inhibition prevents LVD and myocardial remodeling in diabetic and non-diabetic animals exhibiting elevated DPP4 cardiac activity [18,19]. Interestingly, increased plasma DPP4a has been found to be associated with LVD in animals [19,20] and patients [18,19] with HF.

Since there is no available data concerning the activity of circulating DPP4 (DPP4a) and subclinical LVD in patients with T2DM, the aim of this study was to investigate whether plasma DPP4a is associated with echocardiographic parameters assessing subclinical LVDD and/or LVSD in these patients.

## Methods

### Study population

Eighty-three consecutive patients of Caucasian origin, aged >30 years, affected by T2DM, and evaluated at the University of Navarra Clinic between March 2004 and August 2005, were considered for inclusion in this retrospective cross-sectional study. The presence of diabetes was re-evaluated according to the American Diabetes Association criteria [21] (glycated haemoglobin [HbA1c] ≥ 6.5% or fasting glucose ≥ 126 mg/dl or 2 h plasma glucose ≥ 200 mg/dl during an OGTT or use of hypoglycemic medication). All of them were free from clinically apparent cardiovascular disease. Coronary and valve heart disease were excluded by electrocardiographic and echocardiographic evaluation at rest, and by exercise/scintigraphy/echo-stress test.

Fifty-nine age- and sex-matched Caucasian patients coming to the University of Navarra Clinic for a routine medical work-up were included as controls. None of them presented medical history of DM or clinically apparent cardiovascular disease.

According to institutional guidelines, all participants gave written informed consent to participate. The study was carried out in accordance with the Helsinki Declaration and the Ethical Committee of the University of Navarra Clinic approved it.

### Definitions

Concomitant hypertension was defined as systolic blood pressure (SBP) of ≥ 140 mmHg and/or diastolic blood pressure (DBP) of ≥ 90 mmHg and/or the presence of previous chronic antihypertensive treatment. Hypercholesterolemia was diagnosed if the fasting serum total cholesterol was ≥ 200 mg/dL and hypertriglyceridemia was diagnosed if serum triglyceride levels were ≥ 150 mg/dL. Obesity was defined as body mass index (BMI) ≥ 30 kg/m^2^. Estimated glomerular filtration rate (GFR) was determined using the abbreviated Modification of Diet in Renal Disease (MDRD) equation and the urinary albumin-to-creatine ratio was measured. Chronic kidney disease (CKD) was diagnosed if GFR < 60 ml/min/1.73 m^2^ and/or microalbuminuria was present defined as albumin-to-creatinine ratio between 30 and 300 mg/g.

### Echocardiographic study

Two-dimensional echocardiographic imaging, targeted M-mode recordings and Doppler ultrasound measurements were obtained in each patient. LV end-diastolic and systolic volumes (LVEDV and LVESV, respectively), interventricular septum thickness (IVST), posterior wall thickness (PWT) and relative wall thickness (RWT) were calculated as previously reported [22]. RWT > 0.42 was considered indicative of LV concentric remodeling [23]. LVEDV and LVESV were indexed by body surface area (BSA) (LVEDVi and LVESVi, respectively). LV mass index (LVMI) was calculated by dividing LVM by BSA. The presence of LV hypertrophy (LVH) was established when LVMI was above 115 g/m^2^ in men and above 95 g/m^2^ in women in accordance with the American Society of Echocardiography’s Guidelines [23]. The value of LVM directly measured from echocardiograms was divided by that predicted by an equation to predict compensatory LVM as previously described [22] and LVM was expressed as a percentage of predicted, representing the excess relative to the “compensatory” value (i.e. 100% of predicted). Inappropriate LVM was defined as more than 128% of the predicted value as previously described [22]. LV growth was established if LV concentric remodeling and/or LV hypertrophy and/or inappropriate LVM were present. Two-dimensional estimation of left atrial volume (LAV) was performed at LV end-systole. LAV was indexed to body surface area (LAVi). LA enlargement was defined as LAVi ≥ 29 mL/m^2 ^[23].

The following pulsed Doppler measurements of the mitral flow were obtained: maximum early transmitral flow velocity in diastole (E), maximum late transmitral velocity flow in diastole (A), the deceleration time of the early mitral filling wave (DT), and isovolumic relaxation time (IVRT). Tissue Doppler imaging of the lateral mitral annulus was used for measuring the early (e’) and late (a’) mitral annulus velocities throughout the cardiac cycle. Echocardiography evidence of LVDD was established if E/e’ ratio >15. If E/e’ was between 8-15, values of e’ < 9 cm/s and mitral inflow E/A ratio age-corrected abnormal values [24] were considered. In that case, the presence of at least two abnormal measurements was taken into account in order to increase the likelihood of LVDD diagnosis following the European Society of Cardiology’s Guidelines [25].

LV stroke work was calculated as the product of stroke volume and end-systolic pressure as previously described [22]. As a measure of contractility, LV stroke index (LVSWi) was calculated as LV stroke work divided by LVEDV, as previously described [26]. LVEF and subendocardial fractional shortening (FS) were calculated as previously reported [22]. Midwall fractional shortening (MFS) was calculated in accordance with the American Society of Echocardiography’s Guidelines [23]. Circumferential end-systolic stress (cESS) was calculated as previously described [22], to correct MFS (cESS corrected-MFS). mESS was calculated as previously reported [22], and corrected by the LVESVi (mESS/LVESVi). Evidence of LVSD was determined if LVEF < 50%. If LVEF was between 50-55%, LVSD was considered if MFS was lower than 15% in women and 14% in men following the American Society of Echocardiography’s Guidelines [23]. Finally, evidence of LVD was considered if either LVDD and/or LVSD were present.

### Biochemical determinations

Venous blood samples were withdrawn from the left antecubital vein at the time of the clinical studies and stored at -40°C. Plasma DPP4a was measured in duplicate by using the DPP4-Glo™ Protease Assay (Promega). A reference standard curve was measured in each different run by using a purified DPP4 enzyme (BPS Bioscience) with known activity. DPP4a was measured in the absence or the presence of valine pyrrolidide, a specific DPP4 inhibitor, to test the specificity of the enzymatic assay. In our samples, valine pyrrolidide inhibited the assayed activity by >95%. The intra-assay and inter-assay coefficients of variation were 1.86% and 9.95%, respectively and the sensitivity of the technique has been established as the detection of the cleaving activity of 0.5 ng of recombinant DPP4.

Amino-terminal pro-brain natriuretic peptide (NT-proBNP) was measured in serum samples by ELISA (Biomedica Gruppe). The sensitivity was 5 fmol of NT-proBNP/mL. The inter-assay and intra-assay coefficients of variation were lower than 10%.

### Statistical analysis

Continuous variables were reported as mean values ± one standard deviation or, if not normally distributed, as median and interquartile range, whereas categorical variables were reported as numbers and percentages. Differences between non-diabetic subjects and diabetic patients were tested by Student’s *t*-test for unpaired data once normality was demonstrated (Kolmogorov-Smirnov test); otherwise, a nonparametric test (Mann-Whitney *U*-test) was used. Differences in continuous variables between more than two groups were tested by one-way ANOVA followed by a Student-Newman-Keuls test once normality was checked (Shapiro-Wilks test); otherwise, the nonparametric Kruskal-Wallis test followed by a Mann-Whitney *U* test (adjusting the α-level by Bonferroni inequality) was used. Categorical variables were analysed by the χ^2^ test or Fisher’s exact test when necessary. Multiple regression analyses were performed to assess the independent relationship between circulating DPP4a and echocardiographic parameters of LV systolic and diastolic function after adjustment for relevant covariates: age, sex, HbA1c, SBP, presence of CKD, anti-hypertensive treatment and anti-diabetic treatment. Logistic regression analysis was performed to derive odds ratio and 95% confidence intervals adjusted for covariates. Statistical significance was defined as two-sided p < 0.05. The statistical analysis was done using the SPSS software (15.0 version; SPSS Inc., Chicago, Illinois, USA).

## Results

### Clinical characteristics

The demographic and clinical parameters evaluated in non-diabetic subjects and in patients with T2DM are presented in Table 1. As compared with non-diabetics, T2DM patients exhibited higher body mass index (BMI), and decreased diastolic and mean blood pressure values. As expected, the percentage of HbA1c and the fasting glucose levels in blood were significantly increased in T2DM patients as compared with non-diabetic subjects. In addition, the presence of hypertension was similar in both groups although the prevalences of hypercholesterolemia and obesity were lower and higher, respectively, in patients with T2DM than in non-diabetic subjects. As expected, more patients in the diabetic group were under treatment with cardiovascular drugs (including anti-hypertensive medications) than in the non-diabetic group.

**Table 1:** Demographic and clinical parameters in the population according to the presence or absence of diabetes

**Parameters**	**Non-diabetic subjects**	**Diabetic patients**	**p value**
	**(n = 59)**	**(n = 83)**	
Age (years)	63.6 ± 9.5	65.4 ± 8.4	0.214
Male/female (n, %)	33/26, 56/44	52/31, 63/37	0.264
BMI (Kg/m^2^)	28 ± 4.3	29.8 ± 5	0.049
SBP (mmHg)	143 ± 22	139 ± 21.4	0.333
DBP (mmHg)	80 (72-90)	75 (70-80)	0.002
MBP (mmHg)	102 ± 13.9	96.5 ± 12.3	0.016
PP (mmHg)	62.3 ± 17.5	64 ± 19.4	0.605
HbA1c (%)	5.3 (5.1-5.4)	6.7 (5.9-7.5)	<0.001
Fasting glucose (mg/dL)	92 (87-95)	115 (101-138)	<0.001
Comorbidities, (n, %)			
Hypertension	41, 70	68, 82	0.064
Hypertriglyceridemia	8, 14	18, 22	0.158
Hypercholesterolemia	34, 58	23, 28	<0.001
Obesity	18, 30	39, 47	0.049
CKD	5, 8.5	15, 18	0.115
Treatment, (n, %)			
Antidiabetic agents			
Metformin	0, 0	25, 30	
Sulfonylureas	0, 0	18, 22	
Other oral anti-diabetic drugs	0, 0	22, 27	
Insulin	0, 0	23, 28	
Anti-hypertensive agents			
ACEi/ARAs	16, 28	49, 59	<0.001
Diuretics	6, 10	20, 24	0.030
Ca2 + -antagonists	10, 17	19, 23	0.274
Beta-blockers	5, 9	20, 24	0.014
Other pharmacological agents			
Statins	14, 24	48, 58	<0.001
Anti-coagulants	0, 0	14, 16	
Anti-aggregants	14, 24	44, 53	<0.001

BMI means body mass index; SBP, systolic blood pressure; DBP, diastolic blood pressure; MBP, mean blood pressure; PP, pulse pressure; HbA1c, glycosylated haemoglobin; CKD, chronic kidney disease; ACEi, angiotensin converting enzyme inhibitor; ARA, angiotensin II type 1 receptor antagonist. Obesity was defined as BMI ≥ 30 kg/m^2^ and CKD was diagnosed if the estimated glomerular filtration rate was < 60 ml/min/1.73m^2^ and/or microalbuminuria was present defined as an albumin-to-creatinine ratio between 30 and 300 mg/g. Values are expressed as mean ± SD or median (interquartile range), and categorical variables as numbers and percentages.

### Echocardiographic parameters

Table 2 shows the echocardiographic parameters assessed in the population according to the presence or absence of T2DM. Compared with non-diabetic subjects, T2DM patients exhibited higher prevalence of LV concentric remodeling and inappropriate LVM. The prevalence of LVH and LA enlargement was similar in the 2 groups of subjects. In addition, parameters assessing LV diastolic and systolic function were altered in T2DM patients as compared with non-diabetic subjects. Therefore, the prevalence of LVDD and LVSD was higher in patients with T2DM than in non-diabetic subjects. Finally, the prevalence of LVD (considered as the presence of LVDD and/or LVSD) was increased in T2DM patients as compared with non-diabetic subjects (44.6% vs 6.8%, p < 0.001).

**Table 2:** Echocardiographic parameters in the population according to the absence or presence of diabetes

**Parameters**	**Non-diabetic subjects**	**Diabetic patients**	**p value**
	**(n = 59)**	**(n = 83)**	
LV Morphology			
LVEDVindex (mL/m^2^)	64.9 ± 14.8	63.9 ± 15.3	0.714
LVESVindex (mL/m^2^)	22.5 ± 7.8	23.1 ± 8.6	0.695
IVSTd (mm)	10 (8-11)	10 (9-12)	0.033
PWTd (mm)	10 (9-12)	11 (10-12)	0.448
RWT	0.40 ± 0.06	0.44 ± 0.08	0.026
*Prevalence of LV concentric geometry* (n, %)	21, 36	49, 59	0.008
LVM/BSA (g/m^2^)	94.3 (80.1-126)	105 (87.8-127)	0.228
*Prevalence of LVH (n, %)*	27, 46	37, 44	0.510
Observed/predicted LVM (%)	118 ± 31	133 ± 34.7	0.025
*Prevalence of inappropriate LVM (n, %)*	16, 27	43, 52	0.007
LA Morphology			
LA long-axis (cm)	4.9 ± 0.7	5.2 ± 0.8	0.021
LA minor-axis (cm)	3.7 ± 0.7	3.8 ± 0.7	0.385
LA ap (cm)	3.6 ± 0.7	3.7 ± 0.8	0.448
LA volume index (mL/m^2^)	19.2 (14.6-25.1)	18.1 (14.9-27.6)	0.863
*Prevalence of LA enlargement (n,%)*	7, 12	19, 23	0.093
LV diastolic function			
E (cm/s)	74.6 ± 15.2	78.1 ± 20.1	0.353
A (cm/s)	84.3 ± 18.9	88.4 ± 19.5	0.241
E/A ratio	0.91 ± 0.23	0.83 ± 0.16	0.042
IVRT (ms)	110 (90-121)	100 (90-120)	0.113
DT (ms)	220 (180-258)	220 (190-260)	0.811
e' (cm/s)	9.1 ± 2.5	8.3 ± 2.4	0.151
E/e'	8.5 (6.6-10.5)	9.3 (7.6-12)	0.046
a' (cm/s)	10.4 ± 2.8	11.4 ± 3	0.088
e'/a'	0.74 (0.58-1.2)	0.66 (0.54-0.78)	0.028
*Prevalence of LV diastolic dysfunction (n, %)*	3, 5.2	30, 36.6	<0.001
LV systolic function			
LVSWi (g/cm-^2^)	85.2 ± 13.8	78.9 ± 15.6	0.017
LVEF (%)	65.1 ± 6.7	62.2 ± 6.2	0.010
FS (%)	35.9 ± 5.1	33.5 ± 4.8	0.007
MFS (%)	16.8 ± 3	15.8 ± 2.6	0.045
cESS (kdyne/cm^2^)	142 (117-176)	139 (118-175)	0.854
mESS (kdyne/cm^2^)	77.3 ± 18.5	73.1 ± 20.2	0.213
cESS-MFS (%)	99.9 ± 18	94.1 ± 14.8	0.042
mESS/LVESVi (10^7^ dyne/cm^3^)	3.3 (2.4-4.8)	2.9 (2.1-2.9)	0.071
*Prevalence of LV systolic dysfunction (n, %)*	4, 6.8	17, 20.5	0.019

LV means left ventricular; LVEDVi, LV end-diastolic volume index; LVESVi, LV end-systolic volume index; IVSTd, interventricular septum thickness in diastole; PWTd, posterior wall thickness in diastole; RWT, relative wall thickness; LVM, LV mass; BSA, body surface area; LVH, left ventricular hypertrophy; LA, left atrial; E, maximum early transmitral velocity in diastole; A, maximum late transmitral velocity in diastole; IVRT, isovolumic relaxation time; DT, deceleration time; e’, early mitral annulus velocity; a’, late mitral annulus velocity; LVSWi, LV stroke work index; LVEF, LV ejection fraction; FS, subendocardial fractional shortening; MFS, midwall fractional shortening; cESS, circumferential end-systolic stress; mESS, meridional end-systolic stress. Values are expressed as mean ± SD or median (interquartile range), and categorical variables as numbers and percentages.

### Biochemical parameters

Plasma DPP4a was higher in T2DM patients as compared with non-diabetic subjects (5208 ± 957 vs 5855 ± 1632 pmol/min/mL, p < 0.05). In addition, compared with non-diabetic subjects, patients with T2DM exhibited higher levels of NT-proBNP (234 ± 136 vs 348 ±180 fmol/mL, p < 0.01).

### Plasma DPP4a and clinical and echocardiographic characteristics in patients with T2DM

Table 3 shows the clinical features of patients with T2DM classified according to tertiles of plasma DPP4a. Age, gender, BMI, blood pressure, HbA1c, fasting glucose, comorbidities and treatment were similar among the three groups of patients.

**Table 3:** Demographic and clinical parameters according to tertiles of circulating DPP4 activity in diabetic patients

**Parameters**	**DPP4 (pmol/min/mL)**			**p value**
	**<5060**	**5060-6208**	**>6208**	
Age (years)	65 ± 9.1	66.2 ± 7.1	64.7 ± 9.7	0.836
Male/female (%)	59/41	68/32	61/39	0.657
BMI (Kg/m^2^)	32 ± 6.4	28.5 ± 4.5	29.4 ± 4.5	0.086
SBP (mmHg)	140 ± 17	136 ± 23.8	141 ± 23.9	0.767
DBP (mmHg)	80 (70-80)	70 (60-80)	75 (70-80)	0.300
MBP (mm Hg)	97.8 ± 8	93.6 ± 13.8	97.8 ± 13.4	0.395
PP (mmHg)	63.3 ± 17.5	64.1 ± 18.4	64.3 ± 23.2	0.984
HbA1c (%)	6.5 (6.1-7.4)	6.5 (5.9-7.2)	6.9 (6-7.9)	0.555
Fasting glucose (mg/dL)	112 (100-129)	113 (106-169)	117 (109-171)	0.265
Comorbidities, (n, %)				
Hypertension	22, 82	22, 79	24, 85	0.997
Hypertriglyceridemia	5, 19	6, 21	7, 25	0.811
Hypercholesterolemia	4, 15	9, 32	10, 36	0.440
Obesity	16, 60	9, 32	14, 50	0.230
CKD	6, 22	5, 17	4, 14	0.409
Treatment (n, %)				
Antidiabetic agents				
Metformin	9, 33	4, 14	12, 43	0.064
Sulfonylureas	7, 26	7, 26	4, 14	0.199
Other oral anti-diabetic drugs	5, 19	7, 26	10, 36	0.418
Insulin	6, 22	7, 25	10, 36	0.664
Anti-hypertensive agents				
ACEi/ARAs	17, 63	15, 53	17, 61	0.634
Diuretics	6, 22	7, 25	7, 25	0.938
Ca2 + -antagonists	3, 11	9, 32	7, 25	0.104
Beta-blockers	7, 26	7, 25	6, 21	0.888
Other pharmacological agents				
Statins	15, 55	18, 64	15, 57	0.824
Anti-coagulants	5, 19	4, 14	5, 19	0.910
Anti-aggregants	15, 56	15, 53	14, 50	0.902

BMI means body mass index; SBP, systolic blood pressure; DBP, diastolic blood pressure; MBP, mean blood pressure; PP, pulse pressure; HbA1c, glycosylated haemoglobin; CKD, chronic kidney disease; ACEi, angiotensin converting enzyme inhibitor; ARA, angiotensin II type 1 receptor antagonist. Values are expressed as mean ± SD or median (interquartile range), and categorical variables as numbers and percentages.

As shown in Table 4, parameters of LV and LA morphology did not change across the three groups of T2DM patients categorized according to plasma DPP4a.

**Table 4:** Echocardiographic parameters according to tertiles of circulating DPP4 activity in diabetic patients

**Parameters**	**DPP4 (pmol/min/mL)**			**p value**
	**<5060**	**5060-6208**	**>6208**	
LV Morphology				
LVEDVindex (mL/m^2^)	63.7 ± 13	66.4 ± 12	63.9 ± 17	0.794
LVESVindex (mL/m^2^)	20.8 ± 6.6	22.9 ± 9.5	25.4 ± 7.7	0.080
IVSTd (mm)	10 (9-12)	11(9-13)	10 (9-12)	0.450
PWTd (mm)	10 (9-12)	11 (10-12)	11 (9.3-12)	0.095
RWT	0.42 ± 0.06	0.46 ± 0.05	0.42 ± 0.09	0.086
*Prevalence of LV concentric geometry (n, %)*	15, 55	20, 71	14, 50	0.128
LVM/BSA (g/m^2^)	94.6 (81-132)	112 (94.6-134)	110 (84-131)	0.201
*Prevalence of LVH (n, %)*	10, 37	16, 57	11, 40	0.294
Observed/predicted LVM (%)	124 ± 27.9	132 ± 25.4	134 ± 30.6	0.535
*Prevalence of inappropriate LVM* (*n,* %)	13, 48	16, 57	14, 50	0.932
LA Morphology				
LA long-axis (cm)	5.4 ± 0.7	5.1 ± 0.9	5.3 ± 0.8	0.443
LA minor-axis (cm)	4 ± 0.7	3.7 ± 0.7	3.9 ± 0.8	0.605
LA ap (cm)	3.8 ± 0.6	3.5 ± 0.9	3.8 ± 0.9	0.424
LA volume index (mL/m^2^)	20.3 (16-29.3)	17 (14.7-23.7)	20.7 (13.2-33)	0.450
*Prevalence of LA enlargement (n,%)*	7, 26	4, 14	8, 29	0.442

LV means left ventricular; LVEDVi, LV end-diastolic volume index; LVESVi, LV end-systolic volume index; IVSTd, interventricular septum thickness in diastole; PWTd, posterior wall thickness in diastole; RWT, relative wall thickness; LVM, LV mass; BSA, body surface area; LVH, left ventricular hypertrophy; LA, left atrial. Values are expressed as mean ± SD or median (interquartile range), and categorical variables as numbers and percentages.

**Figure 1:**
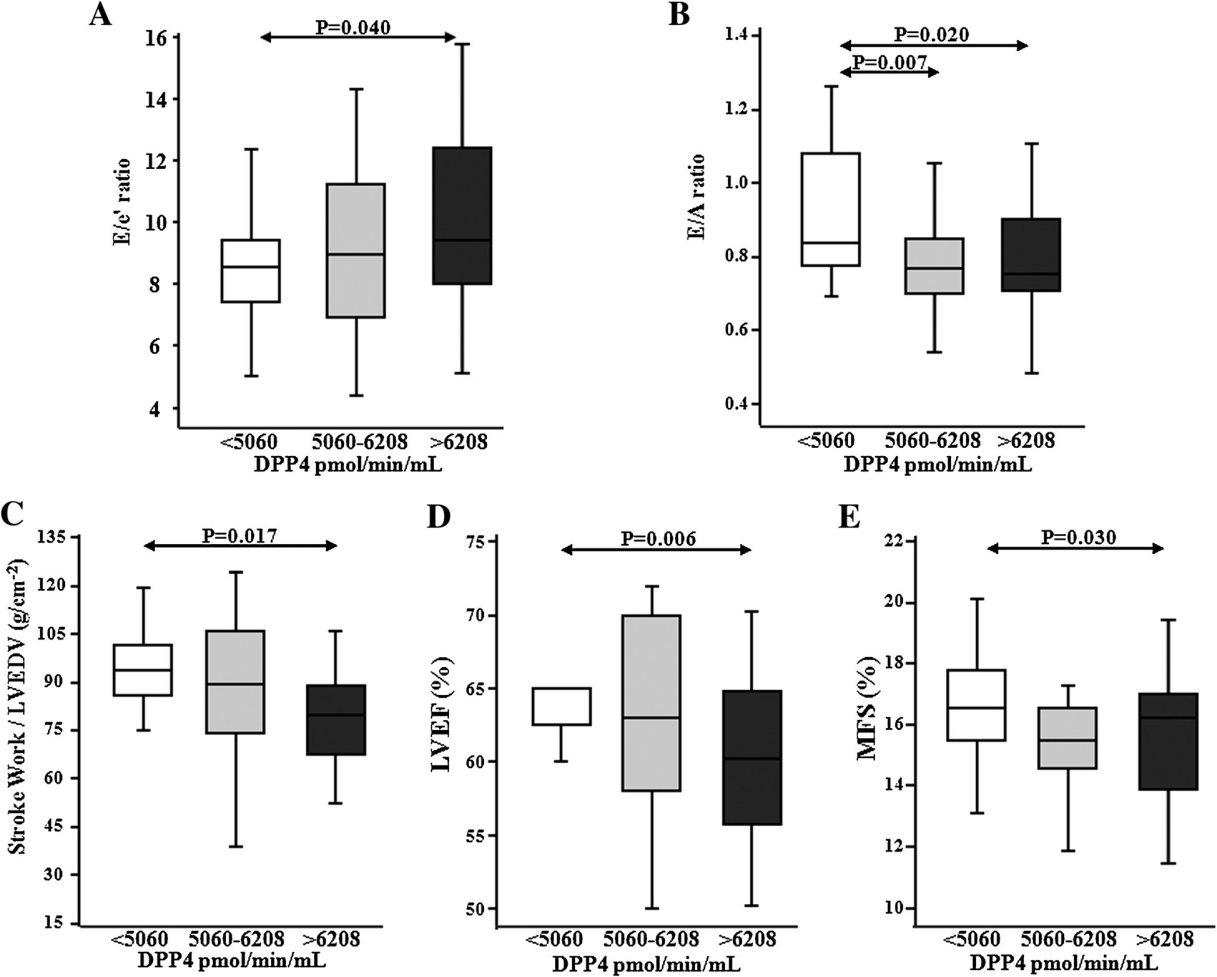
**Distribution of echocardiographic parameters assessing left ventricular diastolic and systolic function in patients with T2DM categorized according to plasma DPP4 activity levels.** Box plots show the 5th and 95th (vertical lines), 25th and 75th (boxes) and 50th (horizontal line) percentile values for E/e’ ratio **(panel A)**, E/A ratio **(panel B)**, stroke work corrected by left ventricular (LV) end-diastolic volume (LVEDV) **(panel C)**, LV ejection fraction (LVEF) **(panel D)** and midwall fractional shortening (MFS) **(panel E)**.

**Figure 2:**
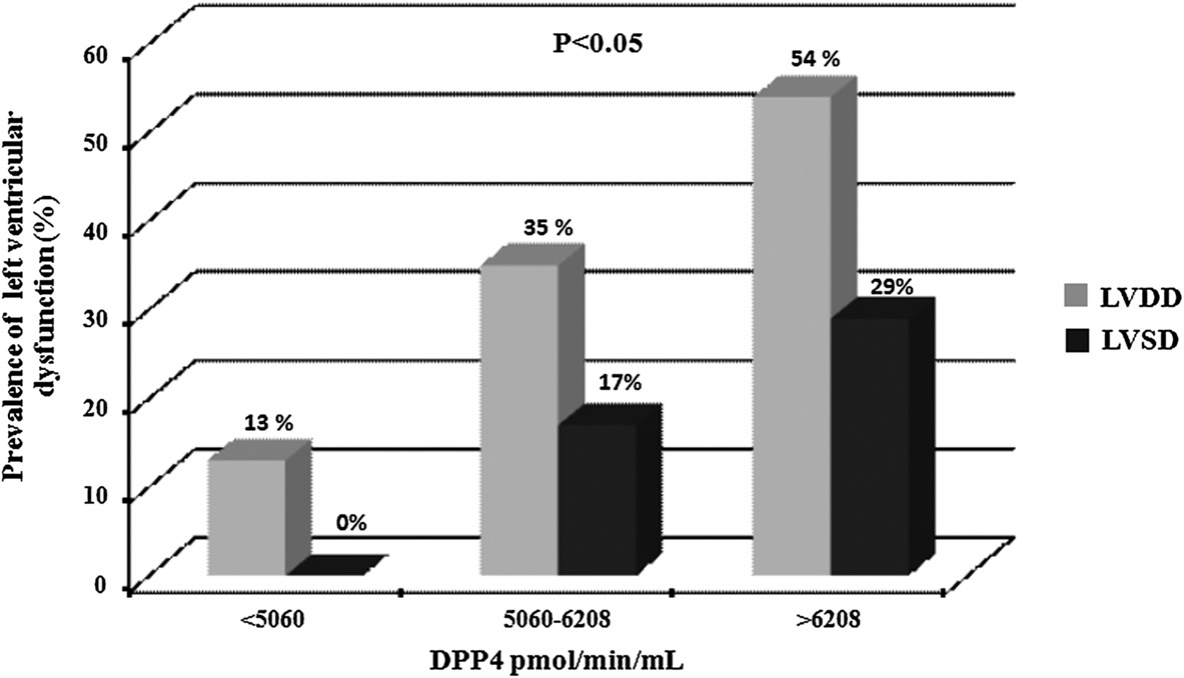
**Prevalence of left ventricular diastolic dysfunction and left ventricular systolic dysfunction in patients with T2DM categorized according to plasma DPP4 activity levels.** Grey bars show the prevalence of left ventricular diastolic dysfunction (LVDD: χ2 = 8.82, p = 0.012) and black bars show the prevalence of left ventricular systolic dysfunction (LVSD: χ2 = 7.62, p = 0.022).

Interestingly, T2DM patients with the highest values of plasma DPP4a (third tertile) exhibited increased values of E/e’ ratio as compared with patients showing lower DPP4a (first tertile) (Figure 1A). In addition, T2DM patients in the second and third DPP4a tertiles showed lower values of E/A ratio than patients in the first DPP4a tertile (Figure 1B). Moreover, T2DM patients in the third tertile of DPP4a had lower values of LVSWi (Figure 1C), LVEF (Figure 1D) and MFS (Figure 1E) as compared with patients in the first tertile. In accordance with these differences, the prevalence of both LVDD and LVSD progressively increased across tertiles of plasma DPP4a in diabetic patients (Figure 2). Finally, the prevalence of LVD was 13%, 39% and 71% in patients from the first, second and third tertiles of plasma DPP4a, respectively (χ^2^ = 16.2, p < 0.001).

NT-proBNP levels did not change across the three tertiles of DPP4 activity (first to third DPP4 tertiles: 338 ± 196 vs 345 ± 180 vs 372 ± 191 fmol/mL, p = 0.823).

### Study of associations

Associations between plasma DPP4a and parameters of LV diastolic and systolic function were analyzed in T2DM patients by multivariate linear regression analysis. After adjusting for potential confounding factors (age, gender, HbA1c, SBP, presence of CKD, anti-hypertensive treatment and anti-diabetic treatment), plasma DPP4a was directly correlated with the E/e’ ratio (β = 0.307, p = 0.036). In addition, an inverse correlation was detected between plasma DPP4a and the E/A ratio independently of gender, HbA1c, SBP, presence of CKD, anti-hypertensive treatment and anti-diabetic treatment (β = -0.295, p = 0.036). However, the multiple regression analysis evaluating the association between plasma DPP4a and the E/A ratio rendered a non-significant β coefficient after adjustment for age. Furthermore, plasma DPP4a was inversely associated with LVSWi (β = -0.210, p = 0.025), LVEF (β = -0.291, p = 0.040) and MFS (β = -0.365, p = 0.008) independently of all the considered potential confounding factors (age, gender, HbA1c, SBP, presence of CKD, anti-hypertensive treatment and anti-diabetic treatment).

No associations of NT-proBNP levels with parameters assessing LV systolic and diastolic function were identified in T2DM patients.

Multiple logistic regression analysis confirmed the previous observations since an increase of 100 pmol/min/mL in plasma DPP4a was significantly associated with a higher risk of LVDD (Table 5) and of LVSD (Table 6) in T2DM patients, independently of all the above considered confounding factors. Thus, an increase of 100 pmol/min/min plasma DPP4a was independently associated with an increased frequency of LVD with an adjusted odds ratio of 1.10 (95% CI, 1.04 to 1.15, p = 0.001). By the same token, T2DM patients in the third tertile of plasma DPP4a had an adjusted odds ratio for LVD of 8.18 (95% CI, 2.19 to 30.6, p = 0.002).

**Table 5:** Multiple logistic regression analysis (dependent variable, presence of left ventricular diastolic dysfunction)

**Significant correlates**	**Units of increase**	**Odds ratio**	**95% CI**	**p value**
DPP4	100 pmol/min/mL	1.05	1.01-1.09	0.027
Not significant correlates				
Age	1 year	1.09	0.99-1.19	0.069
Male gender		0.40	0.10-1.55	0.183
HbA1c	1%	1.09	0.62-1.91	0.776
SBP	1 mm Hg	1.01	0.97-1.04	0.741
CKD	0 = no; 1 = yes	2.30	0.34-15.8	0.396
Anti-hypertensive treatment	0 = no; 1 = yes	0.68	0.14-3.38	0.637
Anti-diabetic treatment	0 = no; 1 = yes	2.01	0.40-10.6	0.413

DPP4 means dipeptidyl peptidase-4; HbA1c, glycosylated haemoglobin; SBP, systolic blood pressure; CKD, chronic kidney disease. Data are expressed as odds ratio and 95% confidence interval.

**Table 6:** Multiple logistic regression analysis (dependent variable, presence of left ventricular systolic dysfunction)

**Significant correlates**	**Units of increase**	**Odds ratio**	**95% CI**	**p value**
DPP4	100 pmol/min/mL	1.10	1.01-1.21	0.032
SBP	1 mm Hg	0.91	0.84-0.99	0.022
Not significant correlates				
Age	1 year	1.11	0.92-1.34	0.264
Male gender		4.45	0.37-54	0.241
HbA1c	1%	0.77	0.24-2.47	0.659
CKD	0 = no; 1 = yes	2.64	0.08-83.4	0.581
Anti-hypertensive treatment	0 = no; 1 = yes	2.86	0.10-80.6	0.538
Anti-diabetic treatment	0 = no; 1 = yes	0.30	0.01-7.31	0.449

DPP4 means dipeptidyl peptidase-4; HbA1c, glycosylated haemoglobin; SBP, systolic blood pressure; CKD, chronic kidney disease. Data are expressed as odds ratio and 95% confidence interval.

## Discussion

The main findings of this study are the following: (1) the activity of circulating DPP4 is abnormally increased in patients with T2DM; (2) increased activity of circulating DPP4 is independently associated with asymptomatic LVDD and LVSD in T2DM patients; and (3) T2DM patients with increased activity of circulating DPP4 exhibit a higher risk of presenting LVD independently of the presence of confounding factors.

In accordance with previous studies [27-29], we show that plasma DPP4a is increased in patients with T2DM. Although it has been reported that plasma DPP4a is associated with HbA1c levels in T2DM patients [27,30], no associations were found between plasma DPP4a and parameters assessing glucose metabolism in this study. On the other hand, although experimental [20] and clinical [31,32] data suggest that overweight and obesity may influence circulating DPP4 levels and activity, no associations of plasma DPP4a with BMI or obesity were found in the current study. Finally, although previous studies have suggested that some oral anti-diabetic agents other than gliptins (e.g., metformin) may alter the activity of circulating DPP4 [33], other studies have failed to reproduce the findings [34]. In our study, plasma DPP4a was independent of the treatment with oral antidiabetic drugs, including metformin, and insulin. Therefore, the mechanisms involved in an excessive activity of circulating DPP4 in T2DM patients from our study remain to be elucidated.

Findings from previous studies show association of the activity of circulating DPP4 with LVD in HF. In fact, experimental studies have shown that increased plasma DPP4a is associated with LVD in animals with HF [19,20]. There are also clinical observations relating an excess of plasma DPP4a with LVD in HF patients, namely in those with diabetes mellitus [18,19]. On the other hand, it has been shown that genetically-induced deficiency of DPP4 or pharmacological inhibition of DPP4 that reduce plasma DPP4a also result in improved LV function. In particular, this has been demonstrated in normoglucemic swine models of ischemia-reperfusion [35] and overpacing-induced HF [36], in normoglucemic rodent models of HF induced by pressure overload [37], radiofrequency LV ablation [19] or myocardial infarction [38], as well as in insulin-resistant obese rodent models [39] and in diabetic rodent models of myocardial infarction-induced HF [40,41]. Collectively, these data suggest that an excessive activity of circulating DPP4 may be related with advanced symptomatic LVD. In this conceptual framework, our study demonstrates for the first time that plasma DPP4a is independently associated with both LVDD and LVSD in asymptomatic T2DM patients, thus suggesting that an excessive activity of circulating DPP4 can be involved in early subclinical LVD in T2DM.

The question arises of which mechanisms link the activity of circulating DPP4 with LVD. One possibility is that DPP4 inactivates circulating peptides that possess cardioprotective actions, including GLP-1, BNP, and peptide YY [42,43]. However, the majority of these substrates serve as pharmacological targets *in vitro*, but few have been shown to be endogenous, physiological substrates (defined as peptides whose endogenous circulating level of intact versus N-terminally cleaved forms is altered after reduction or elimination of DPP4 activity *in vivo*) [44]. An alternative possibility is that increased activity of circulating DPP4 coincides with increased activity of cardiac DPP4, thus allowing for direct detrimental actions of the enzyme on the myocardium. This possibility is based on two observations [18]. First, a direct correlation between DPP4a measured in blood from the antecubital vein and that measured in blood from the coronary sinus has been reported in humans. Second, myocardial DPP4 overactivity was found to be associated with reduced myocardial availability of stromal cell-derived factor 1α and impaired angiogenesis and fibrosis in diabetic rats with HF.

The third finding of this study is that plasma DPP4a is an independent risk factor for subclinical LVD in T2DM patients. In fact, an increase of 100 pmol/min/min plasma DPP4a was independently associated with 10% increase in the risk of subclinical LVD in T2DM patients. It has been recently shown that the likelihood of subclinical LVD in these patients increases independently with age, HbA1c, and treatment with metformin [5]. Interestingly, the greater risk of subclinical LVD associated with plasma DPP4a was independent of these factors. On the other hand, although increased NT-proBNP has been proposed as a risk factor for subclinical LVD in T2DM patients [45,46], no association was found in this study between NT-proBNP levels and subclinical LVD. Therefore, plasma DPP4a emerges as a useful variable to predict early-stage LVD in T2DM patients without known cardiac disease. The potential clinical relevance of this possibility is given by the high prevalence of subclinical LVD in patients with T2DM (e.g. almost 45% in our study). Furthermore, considering the high risk for subclinical LVD to evolve to overt HF in T2DM patients [10,11], plasma DPP4a may be also useful as a therapeutic guide to prevent the deterioration of LV function in these patients.

### Limitations

Some limitations of the current study must be recognized. First, data here presented are relevant to a selected sample from a single centre. Second, the cross-sectional design of the study does not allow for causal interpretation of the relationships found. Third, unfortunately, we could not determine concentrations of active (GLP-1 [7-36]), as samples were not collected in tubes with a DPP4 inhibitor. Therefore, future studies should establish how much of plasma DPP4a is related to GLP-1 (7-36) concentration and whether the active GLP-1 peptide may be influencing the associations found between plasma DPP4a and LVD. Fourth, whereas LVSD was assessed using conventional Doppler echocardiography, in accordance with the American Society of Echocardiography’s Guidelines [23], we are aware that more refined methods (e.g. speckle tracking) would allow for a more detailed characterization of LV contraction. Fifth, it must be recognized that inclusion of untreated diabetics and patients treated with different classes of drugs may have confounded the findings here obtained and their interpretation. However, as mentioned above, the pharmacological treatment did not seem to affect the associations of plasma DPP4a with parameters of LV function.

In summary, findings from the current study show that the activity of circulating DPP4 is associated with subclinical LVD in T2DM patients with no coronary or valve heart disease. Albeit preliminary and descriptive in nature, these results set the stage for further experimental studies aimed to test the potential involvement of an excess of DPP4 in the pathogenesis of LVD in diabetes. Furthermore, adequate prospective studies should be considered in order to explore the usefulness of plasma DPP4a as a diagnostic biomarker and a therapeutic target for HF in patients with T2DM. These aspects can be particularly relevant taking into account that, although some meta-analysis indicate that inhibition of DPP4a with gliptins may decrease the risk of HF and other adverse cardiovascular events in T2DM patients [47-49], it has been recently reported that DPP4 inhibition with saxagliptin is associated with increased risk of hospitalization for HF in T2DM patients [50].

## Abbreviations

T2DM: Type 2 diabetes mellitus; DPP4: Dipeptidyl peptidase-4; DPP4a: Plasma DPP4 activity; HF: Heart failure; NT-proBNP: Amino-terminal pro-brain natriuretic peptide; BMI: Body mass index; SBP: Systolic blood pressure; DBP: Diastolic blood pressure; MBP: Mean blood pressure; PP: Pulse pressure; HbA1c: Glycosylated haemoglobin; CKD: Chronic kidney disease; ACEi: Angiotensin converting enzyme inhibitor; ARA: Angiotensin II type 1 receptor antagonist; LV: Left ventricular; LVD: LV dysfunction; LVDD: LV diastolic dysfunction; LVSD: LV systolic dysfunction; LVEDVi: LV end-diastolic volume index; LVESVi: LV end-systolic volume index; IVSTd: Interventricular septum thickness in diastole; PWTd: Posterior wall thickness in diastole; RWT: Relative wall thickness; LVM: LV mass; BSA: Body surface area; LVH: Left ventricular hypertrophy; LA: Left atrial; E: Maximum early transmitral velocity in diastole; A: Maximum late transmitral velocity in diastole; IVRT: Isovolumic relaxation time; DT: Deceleration time; e’: Early mitral annulus velocity; a’: Late mitral annulus velocity; LVSWi: LV stroke work index; LVEF: LV ejection fraction; FS: Subendocardial fractional shortening; MFS: Midwall fractional shortening; cESS: Circumferential end-systolic stress; mESS: Meridional end-systolic stress; 95% CI: 95% confidence interval.

## Competing interests

No potential conflicts of interest relevant to this article were reported.

## Authors’ contributions

SR analyzed the data and wrote the manuscript. ICC enrolled patients and collected data. JB contributed to data collection and supervised the echocardiographic studies. AH, managed data entry and helped research the data. BL and AG, collected samples and helped research the data. JD contributed to discussion, reviewed and edited the manuscript and directed the study. All authors read and approved the final manuscript.
